# Hepatic Abscess

**Published:** 1890-01-18

**Authors:** 


### HEPATIC ABSCESS.

Dr. F. F. Bond, Rastrick, Yorks., publishes a case of "Abscess of the Liver" (*Provincial Medical Journal*) occurring in a boy seven years old, born in India. The early history of the case is not given, but when seen by Dr. Bond, the boy was suffering from acute pain in the lower part of the chest on the right side, with dry cough, and slight rise of temperature. Physical signs were increased dulness over the lower four ribs, diminished resonace, and dry friction murmur. A doubtful diagnosis of pleurisy with effusion, causing secondary liver changes, was made. On a consultant being called in, empyema was diagnosed, and the local application of ung. iodidi recommended. This did little or no good, attacks of fever occurred, and the lower margin of the liver became very hard. Dr. Dolan was consulted, and liver abscess diagnosed. The parents now consenting to operative measures, an aspiration was made, and about two ounces of thick yellow pus drawn off, after which the boy steadily improved. Eleven days afterwards pus had again collected, burst through the site of the puncture, and continued to discharge for some time. Eight or nine days afterwards offensive fœces, pus, and blood were discharged *per rectum*. From this time recovery set in. The author remarks that the case was interesting from the great difficulty of diagnosis, at the beginning the symptoms being identical with those of pleurisy. It was not until the marked local swelling appeared "that it became evident the liver was fault." Now, the difficulty of diagnosis in many cases of liver abscess is well known to Indian practitioners, and such a case in India would probably have been diagnosed as hepatic abscess immediately; but in this country one is more disposed to regard symptoms mentioned as thoracic. Had the parents agreed to an exploratory measure when first proposed, the nature of the malady would have been discovered and the patient would probably have been spared much suffering; but this the parents would not do. Bontius, two hundred years ago, taught that the only chance of recovery from hepatic abscess is to "open the imposthume." The advice still holds good. And the necessity of the procedure has been further demonstrated since Bontius's time by the ill-success which attended the practice most in vogue in the earlier part of the century, of allowing a liver abscess to open spontaneously. If a liver abscess is at all large or deep-seated, it has a tendency to spread laterally, and not to approach the surface. As it spreads it destroys the texture of the liver, until at length nearly the whole of the organ may become one vast abscess sac. A small superficial abscess often comes to the surface. But in any case, when, by aspiration, or by other means, there is reasonable grounds for suspecting the presence of pus, the proper treatment is to evacuate the matter as early as possible.

